# 4-PBA Attenuates Fat Accumulation in Cultured Spotted Seabass Fed High-Fat-Diet via Regulating Endoplasmic Reticulum Stress

**DOI:** 10.3390/metabo12121197

**Published:** 2022-11-30

**Authors:** Tian Xia, Yan-Qin Liao, Lei Li, Lu-Yu Sun, Neng-Shui Ding, You-Lin Wu, Kang-Le Lu

**Affiliations:** 1 Key Laboratory of Healthy Mariculture for the East China Sea, Ministry of Agriculture and Rural Affairs, Fisheries College, Jimei University, Xiamen 361021, China; 2 Fujian Aonong Biological Science and Technology Group Co., Ltd., Zhangzhou 363000, China; 3 Fujian Yixinbao Biopharmaceutical Co., Ltd., Zhangzhou 363000, China

**Keywords:** endoplasmic reticulum stress, high-fat diet, fat deposition, spotted seabass, 4-PBA

## Abstract

Excessive fat accumulation is a common phenomenon in cultured fish, which can cause metabolic disease such as fatty liver. However, the relative regulatory approach remains to be explored. Based on this, two feeding trials were conducted. Firstly, fish were fed either a normal-fat diet (NFD) or a high-fat diet (HFD) for eight weeks and sampled at the 2nd, 4th, 6th, and 8th week after feeding (*Experiment I*). In the first four weeks, fish fed an HFD grew faster than those fed an NFD. Conversely, the body weight and weight gain were higher in the NFD group at the 6th and 8th weeks. Under light and transmission electron microscopes, fat accumulation of the liver was accompanied by an obvious endoplasmic reticulum (ER) swell. Accordingly, the expressions of *atf-6*, *ire-1*, *perk*, *eif-2α*, *atf-4*, *grp78,* and *chop* showed that ER stress was activated at the 6th and 8th weeks. In *Experiment II*, 50 mg/kg 4-PBA (an ERs inhibitor) was supplemented to an HFD; this was named the 4-PBA group. Then, fish was fed with an NFD, an HFD, and a 4-PBA diet for eight weeks. As the result, the excessive fat deposition caused by an HFD was reversed by 4-PBA. The expression of ER stress-related proteins CHOP and GRP78 was down-regulated by 4-PBA, and the transmission electron microscope images also showed that 4-PBA alleviated ER stress induced by the feeding of an HFD. Furthermore, 4-PBA administration down-regulated SREBP-1C/ACC/FAS, the critical pathways of fat synthesis. In conclusion, the results confirmed that ER stress plays a contributor role in the fat deposition by activating the SREBP-1C/ACC/FAS pathway. 4-PBA as an ER stress inhibitor could reduce fat deposition caused by an HFD via regulating ER stress.

## 1. Introduction

Fat plays a key role in the energy supply and storage of animals due to its high calorie level [1]. In fish farming, the use of high-fat diets has become a common practice to achieve a sparing protein effect [2,3]. However, recent studies have indicated that the long-time intake of a high-fat diet often induces excess fat deposition referred to as fatty liver. Then, it can cause metabolic disorders, immune dysfunction, and higher mortality [4,5,6]. Moreover, the substitution of fish oil with plant oil also caused the development of fatty liver and increased the inflammation of fish [7]. Accordingly, it is vital to recognize the mechanisms involved in the development of fatty liver.

The endoplasmic reticulum (ER) is an organelle in eukaryotes vital for protein maturation, and ER stress (ERs) in the liver plays a causative role in the fatty liver of mammals [8]. Additionally, fish fed with a high-fat diet exhibit the disturbance of ER [9]. ER disturbance often leads to misfolded protein accumulation, which induces ERs [10]. In the mice study, ER stress is linked to the development of obesity, and the protein CHOP may be crucial in the interaction between fat deposition and inflammation [11]. It is well-known that fat deposition in the liver represents complex processes. Till now, how ER stress affects the development of fatty liver in fish is still not very clear. Moreover, some therapies acting on ER are suggested as novel strategies for the prevention of fatty liver [12].

Spotted seabass (*Lateolabrax maculatus*) is a carnivorous species with rapid growth speed and a high economic value and thus it has become the second-largest cultivated marine fish in China [13]. For its artificial rearing, the high-fat diet (crude lipid > 15%) is widely used and often leads to the development of fatty liver [3]. Hence, spotted seabass is a good fish model to study the relationship between ER stress and fatty liver. Moreover, we want to explore the role of 4-PBA (an inhibitor of ER stress) in protection against excess fat deposition in fish.

4-PBA is an FDA (The United States Food and Drug Administration)-approved drug currently used for the treatment of urea cycle disorders (UCDs). 4-PBA is an endoplasmic reticulum stress inhibitor that is helpful for protein folding in the endoplasmic reticulum and alleviates the negative effects of endoplasmic reticulum stress [14]. 4-PBA alleviates weight gain and fat production in mice exposed to a high-fat diet by inhibiting endoplasmic reticulum stress [15]. Other studies have shown that 4-PBA can reverse the apoptosis of mouse embryos exposed to high glucose, which is related to endoplasmic reticulum stress [16]. There are many studies on 4-PBA in mammals, which is closely related to endoplasmic reticulum stress. However, there are few studies on the relationship between 4-PBA and endoplasmic reticulum stress and fat deposition in fish. 4-PBA has great potential in green and healthy fish culture.

## 2. Materials and Methods

### 2.1. Fish and Experimental Diets

The animal study protocol was approved by The Committee on the Ethics of Animal Experiments of Jimei University, China (protocol code 2011-58 and approved by 20 December 2011).

A local fish breeding farm (Zhangzhou, Fujian, China) provided juvenile *L. maculatus*. Fish were transported to a recirculating aquaculture system in the laboratory located at Jimei University. These juveniles were maintained in a 1200 L tank for two weeks to acclimatize to the experimental condition. During this period, the fish were fed a commercial diet twice daily (8:00 and 17:00).

Two experimental diets were formulated with a different crude fat level and named normal-fat diet (NFD, 11% fat) and high-fat diet (HFD, 17% fat). A 4-PBA supplementation diet was formulated by adding 4-PBA to the HFD (deemed 4-PBA), at a dose of 50 mg/kg. The feed formulation and nutritional composition can be observed in Supplementary Materials Table S1. The method of feed preparation and nutrient level determination was described in our recent study [13].

### 2.2. Experimental Design

#### 2.2.1. Experiment I: High-Fat-Diet Feeding Trial

A total of 180 healthy fish of a similar size (13.05 ± 0.10 g) were randomly distributed into six tanks (30 fish per tank). Fish were fed the NFD or HFD diets for 8 weeks twice daily (8:00 and 17:00). The light duration was 12 h/d. During the experiment, the fish were fed to satiation. Each treatment had three replicates. Fish were sampled at the 2nd, 4th, 6th, and 8th week of the feeding trial. For each sample time, all fish from each tank were weighed and counted to calculate the average body weight; after that, three fish from each tank were captured randomly and their liver and blood were sampled. The liver samples were put into liquid nitrogen and transferred to −80 °C until analysis. The blood samples were centrifuged (850× *g*, 10 min) at 4 °C, and then the serum was separated and transferred to −80 °C.

#### 2.2.2. Experiment II: Effects of 4-PBA on Fat Deposition

To further determine the effect of ERs in the pathological process of fatty liver, an ERs inhibitor, 4-PBA, was used. Fish from the same batch used in *Expt I* were randomly allocated to three groups (30 fish per tank, three tanks per group, size 41.00 ± 0.01 g). After being fed an NFD, HFD, and 4-PBA diet, respectively, for eight weeks, fish were sampled with the same method used in *Expt I*.

During the feeding trials, water conditions were maintained as below: dissolved oxygen (>6 mg/L), pH (6.9–7.2) kept stable, and the optimum water temperature (25–27 °C). The light duration was 12 h/d. For sampling, fish were fasting for 24 h and euthanized using 100 mg/L MS-222, then measured the body weight for the calculation of growth indices. Then, five fish were randomly selected and captured from each tank for sampling liver and serum, as per our earlier work [17].

### 2.3. Liver Histology

For oil red O staining, liver samples were fixed in 4% paraformaldehyde for 24 h and then dehydrated in a 15% and 30% sugar solution at 4 °C. The dehydrated samples were sliced into 8 μm-thick sections using a cryostat (Cryostar NX50, Thermo, Waltham, MA, USA). The sections were then stained and photographed. Furthermore, the structure of the ER and mitochondria in hepatocytes was examined using transmission electron microscopy (TEM) analysis. The protocol was described in a previous study [4].

### 2.4. Gene Expression

The FastPure Cell/Tissue Total RNA Isolation Kit (Vazyme Biotech Co., Ltd. Nanjing, China) was used to extract the total RNA from the liver. The concentration and purity of isolated RNA were assayed under a spectrophotometer (NanoDrop Technologies, Waltham, MA, USA). The integrity of the RNA was examined through 1% agarose gel electrophoresis, and a NanoDrop™ One spectrophotometer (Thermo Scientific, Waltham, MA, USA) was used to determine the purity at 260/280 nm. Then, the first-strand cDNA was synthesized from 1 μg of each RNA sample using a commercial kit (Vazyme Biotech Co., Ltd. Nanjing, China), and gDNA wiper was performed to remove the remaining genomic DNA. Quantification was performed using a ABI StepOnePlus Real-Time PCR System (Life Technologies, New York, NY, USA).

According to our recent work, real-time quantitative PCR (qPCR) was carried out [18]. The primers were designed with Primer 5.0 software and synthesized by Genewiz Co. Ltd. (Suzhou, China). Table S2 displayed the primer sequences used in this study. The relative expression levels of target genes were normalized by β-actin and calculated by the 2^−ΔΔCt^ method. The amplification efficiency of all primers was verified among 90–110%.

### 2.5. Western Blotting

Total liver protein was extracted using RIPA buffer (Thermo Scientific, Waltham, MA, USA) for the determination of protein expression. In total, 20 μg of total protein was loaded into each well and separated by 12% sodium dodecyl sulfate-polyacrylamide gel electrophoresis, transferred to PVDF membranes, and blocked by 5% (*w/v*) skimmed milk in TBST buffer (LABLEAD Inc., Beijing, China), following primary antibody incubations with anti-CHOP (PA5-88116, Invitrogen, Carlsbad, CA, USA), anti-GRP78 (ab108613, Abcam, Cambridge, UK), or anti-GAPDH (ab181602, Abcam, UK) overnight at 4°C. Then, it was incubated for one hour with the secondary antibody Goat Anti-Rabbit IgG H&L (HRP) pre-absorbed (ab7090, Abcam, Cambridge, UK). The protein bands were detected by ECL Substrate (BIO-RAD, Hercules, California, USA). The protein concentration used for the western blot was 1.5 mg/g. ImageJ 1.44p was used to analyze the gray value of bands [19,20].

### 2.6. Measures of Biochemical Parameters

The contents of triacylglycerols (TG) and total cholesterol (T-CHO) were measured by commercial kits (Nanjing JianCheng Bioengineering Institute, Nanjing, China). TG was measured using the GPO-PAP enzymatic method. T-CHO was measured by the COD-PAP method. The specific assay steps are performed according to the instructions of the commercial kit.

### 2.7. Statistical Analysis

For *Expt I*, the two groups that sampled simultaneously were compared using a student’s *t*-test. For *Expt II*, one-way ANOVA followed by Tukey’s post-hoc test was used to analyze the differences among the three treatments. All of the results were presented as means ± standard errors (SE), and the significance difference level was set at *p* < 0.05 in both *Expt I* and *Expt II*.

## 3. Results

### 3.1. Experiment I: High-Fat-Diet Feeding Trial

#### 3.1.1. Fish Growth and Fat Accumulation

**Figure 1:**
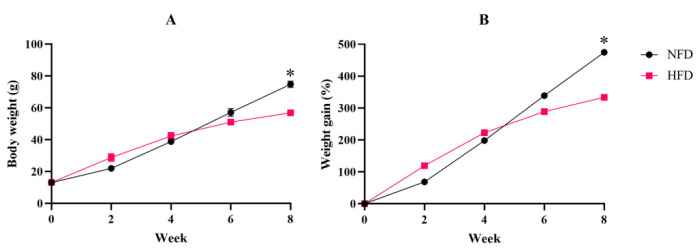
Experiment I. (**A**) Body weight. (**B**) Weight gain. “*” indicates a significant difference between groups (*p* < 0.05, Student’s *t*-test, *n* = 3). Weight gain = final body weight/initial body weight.

The body weight of spotted seabass fed an NFD or HFD was measured at the 2nd, 4th, 6th, and 8th weeks of the feeding trial. In the 8th week, the body weight and cumulative weight gain of the NFD group were significantly higher than that of an HFD (Figure 1A,B).

**Figure 2:**
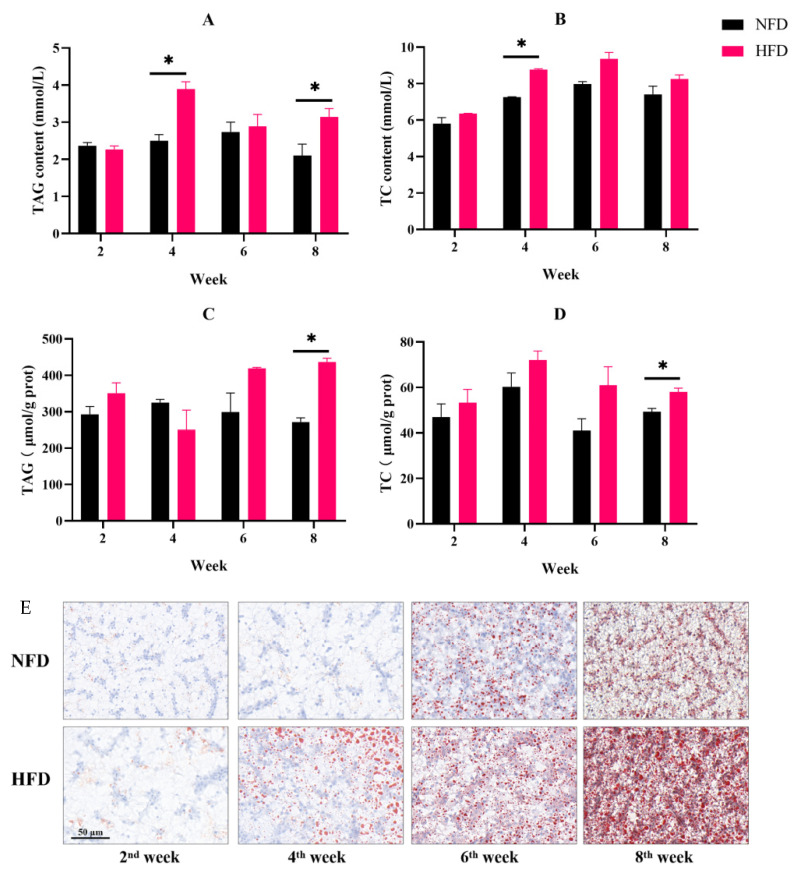
Experiment I. (**A**) Serum TAG content. (**B**) Serum TC content. (**C**) Hepatic TAG content. (**D**) Hepatic TC content. (**E**) Liver oil red O-staining ((**E**) scale bar = 50 μm). “*” indicates a significant difference between groups (*p* < 0.05, Student’s *t*-test, *n* = 3).

In the 4th and 8th weeks, the content of serum triglyceride in the HFD group was significantly higher than that of the NFD group. The serum total cholesterol concentration of fish in the HFD group was considerably greater than the NFD group in the 4th week (Figure 2A,B). In the 8th week, the HFD group’s hepatic triglyceride and total cholesterol contents were noticeably higher than those of the NFD group (Figure 2C,D).

Oil-red O staining was used to evaluate the fat deposition. As a result, hepatic lipid droplets increased over time both in the NFD and HFD groups. However, fish fed an HFD exhibited an enhanced number and a volume of lipid droplets compared to an NFD at the 4th, 6th, and 8th weeks (Figure 2E).

**Figure 3:**
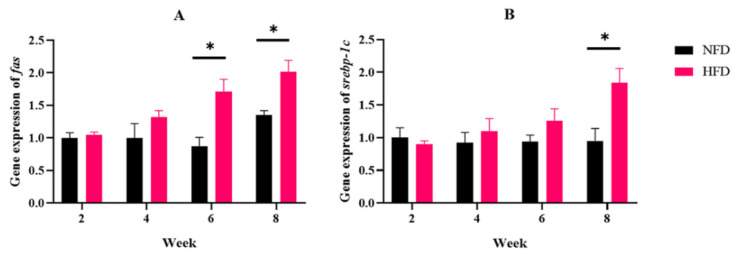
Experiment I. The expressions of fat metabolism-related genes in the liver. (**A**) Gene expression of *fas*. (**B**) Gene expression of *srebp-1c*. “*” indicates a significant difference between groups (*p* < 0.05, Student’s *t*-test, *n* = 3).

Furthermore, the expression of lipid synthesis key genes *fas* and *srebp-1c* in the liver was determined. In the NFD group, both *fas* and *srebp-1c* expressions showed no obvious difference within eight weeks. The expression of *fas* in fish fed an HFD obviously increased after six weeks of feeding and was obviously higher than the fish in the NFD group. Similarly, the HFD group up-regulated *srebp-1c* after eight weeks, and its expression was remarkably higher than in the NFD group (Figure 3A,B).

#### 3.1.2. Ultrastructure of Hepatocytes

**Figure 4:**
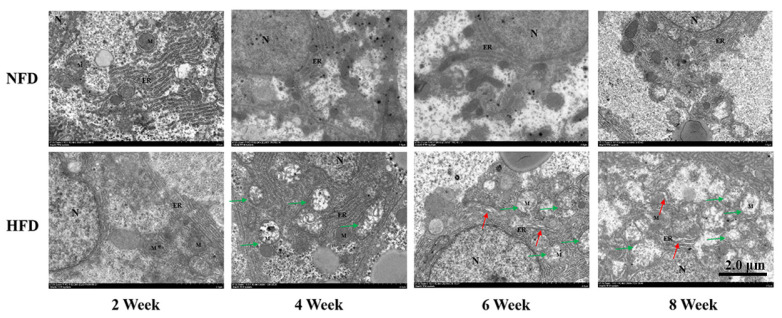
Experiment I. Transmission electron microscopy images of the fish liver at the 2nd, 4th, 6th, and 8th week of feeding trial (*n* = 3). scale bar = 2.0 μm; N, nucleus; ER, endoplasmic reticulum; M, mitochondria. Red arrows: damaged endoplasmic reticulum, green arrows: damaged mitochondria.

The structure of hepatic mitochondria and ER was observed by TEM. The NFD group exhibited a normal ultrastructure during the experimental period. However, after two weeks of feeding, the mitochondrial injury was exhibited in the HFD group. Additionally, the dilated ER was observed after the 6-week feeding of an HFD. Remarkably, swell and cristae lost mitochondria were accompanied by fragmented and expanded ER in the 8th week (Figure 4).

#### 3.1.3. ERs-Related Gene Expressions

**Figure 5:**
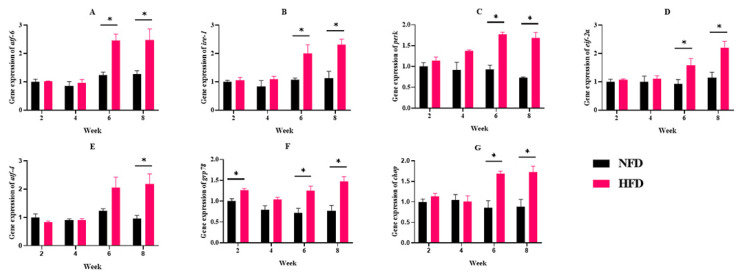
Experiment I. The expressions of endoplasmic reticulum stress-related genes in the liver. (**A**) Gene expression of *atf6*. (**B**) Gene expression of *ire-1*. (**C**) Gene expression of *perk*. (**D**) Gene expression of *eif-2α*. (**E**) Gene expression of *atf4*. (**F**) Gene expression of *grp78*. (**G**) Gene expression of *chop*. “*” indicates a significant difference between groups (*p* < 0.05, Student’s *t*-test, *n* = 3).

Considering that the ER ultrastructure damage was observed, we further determined the expression of ERs-related genes. NFD feeding has no appreciable impact on the expression of the ERs-related genes (*atf-6*, *ire-1*, *perk*, *eif-2α*, *atf-4*, *grp78*, and *chop*). The *atf4* gene expression was significantly up-regulated in the 8th week in the HFD group. In addition, the expression of ERs-related genes (*atf-6*, *ire-1*, *perk*, *eif-2α*, *grp78*, and *chop*) was significantly up-regulated in the HFD group at the 6th and 8th week (Figure 5).

### 3.2. Experiment II: Effects of 4-PBA on Fat Deposition

#### Effects of 4-PBA on Fat Accumulation Caused by an HFD

**Figure 6:**
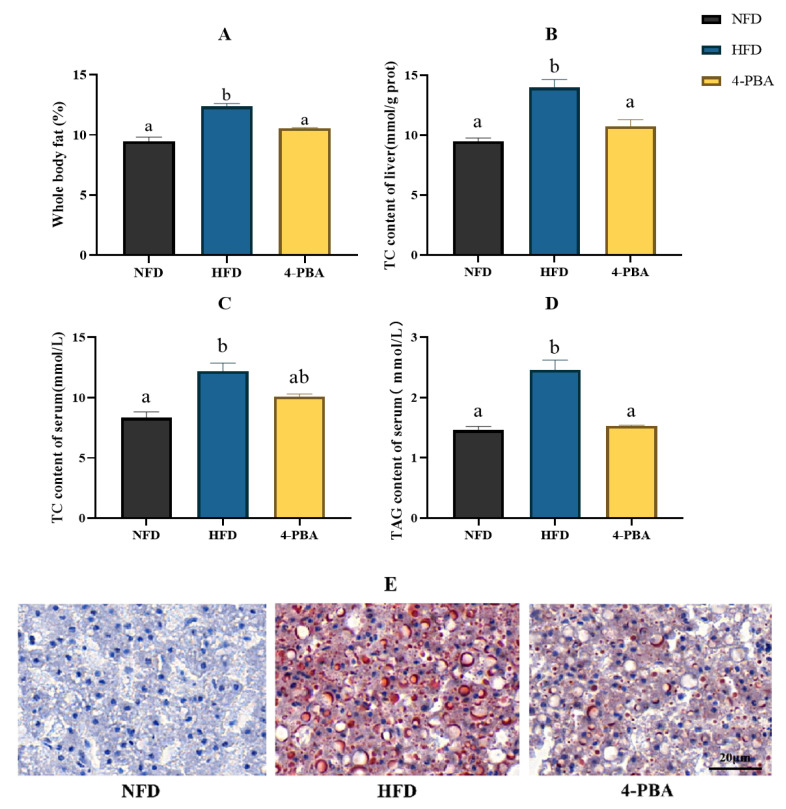
Experiment II. (**A**) Whole body fat. (**B**) Hepatic TC content. (**C**) Serum TC content. (**D**) Serum TAG content. (**E**) Liver oil red O-staining ((**E**) scale bar = 20 μm). All values are exhibited as mean ± SE (*n* = 3). Different letters indicate a significant difference among groups (*p* < 0.05, Tukey’s test, *n* = 3). Whole body fat = abdominal fat weight/whole body weight.

**Figure 7:**
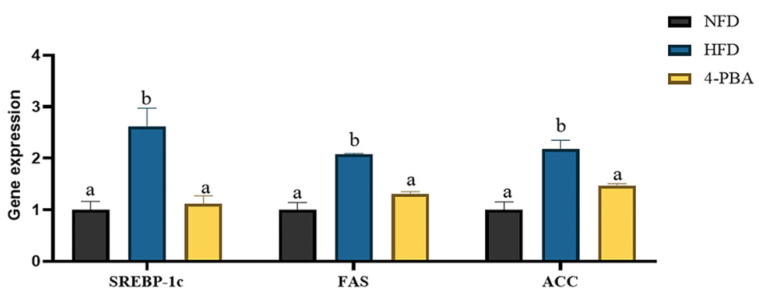
Experiment II. The liver’s expression of genes associated with fat metabolism. Gene expression of *srebp-1c*, *fas*, and *acc*. All values are exhibited as mean ± SE. Different letters indicate a significant difference among groups (*p* < 0.05, Tukey’s test, *n* = 3).

After eight weeks of feeding, the whole body and abdominal fat levels were dramatically raised by an HFD, which were significantly reversed by 4-PBA supplementation. Additionally, the increase in TAG and TC contents in serum and liver induced by an HFD was reversed by the supplementation of 4-PBA (Figure 6A–D). Additionally, fish given HFD had an overabundance of lipid droplets in the liver, shown by oil red O-stained sections. The addition of 4-PBA to an HFD markedly reduced the lipid droplet accumulation (Figure 6E). Accordingly, the expressions of lipid synthesis-related genes *srebp-1c*, *fas*, and *acc* were significantly up-regulated by an HFD while 4-PBA down-regulated their values (Figure 7).

**Figure 8:**
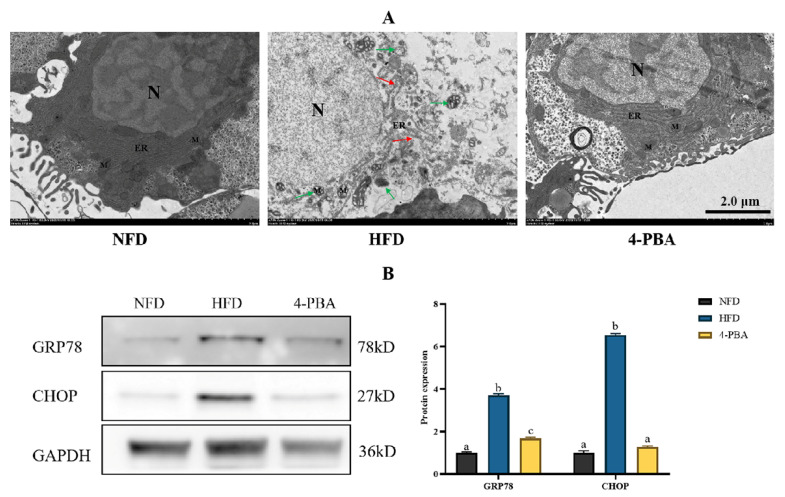
Experiment II. (**A**) Transmission electron microscopy images of fish liver. Scale bar = 2.0 μm. (**B**) GRP78 and CHOP were analyzed through western blotting in the liver of fish. N, Nucleus; ER, endoplasmic reticulum; M, mitochondria. Red arrows: damaged endoplasmic reticulum, green arrows: damaged mitochondria. All values are exhibited as mean ± SE. Different letters indicate a significant difference among groups (*p* < 0.05, Tukey’s test, *n* = 3).

The ER and mitochondria in the NFD group exhibited normal morphology. The ER lumen in the HFD group expanded, and the ER network was ruptured, accompanied by swollen mitochondria. Interestingly, the supplementation of 4-PBA significantly decreased these alterations caused by an HFD (Figure 8A). Furthermore, the HFD group had a higher abundance of CHOP and GRP78, and 4-PBA significantly reduced the expression level of these two proteins (Figure 8B).

## 4. Discussion

In the present, we found that an HFD caused excessive fat deposition through oil red O staining. This was also evidenced by the results of liver TAG and TC contents. Similar results were also reported in some previous in *L. maculatus* [21] and other fish [20,22,23]. The excessive fat deposition was a result of the imbalance of catabolism and anabolism [24,25,26]. ER was found to be an important participator in cellular biosynthesis [27,28]. Hence, there is a tight correlation between fat deposition and ER health. Here, we observed the abnormal ER ultrastructure in fish fed an HFD for more than six weeks. At a similar time point, excessive hepatic fat accumulation is emerging. Similarly, ER damage was found to be accompanied by the fatty liver phenomenon in some previous studies. Collectively, we hypothesize that ER injury may play an important role in the pathological excessive fat deposition caused by long-term HFD feeding.

Recently, the increasing data have demonstrated that ERs was involved in the regulation of ER function and acted as a crucial pathogenic mechanism in metabolic disease. ER is the main location of protein folding, maturation, and secretion. ER dysfunction could be induced by ROS, inflammation-related proteins, and fat overloading, resulting in protein misfolding and unfolding [8]. During these situations, mild or moderate ERs is often regarded as a helpful protective response as the activation of UPR could promote molecular chaperone production, wiping off the unfolded/misfolded proteins [27,29]. However, the long-term energy surplus often creates severe or/and prolonged ERs, meaning that ERs turn out to be detrimental. Accordingly, the results in this study showed that long-term (≥six weeks) HFD feeding caused remarkable up-regulation of *atf6, perk, ire1, grp78,* and *chop*. ATF6, PERK, and IRE1 are the three promoters in the UPR pathway; these pathways could be activated by the release of molecular chaperone GRP78 and induce cell-death signaling via CHOP [30]. Furthermore, the increased GRP78 and CHOP protein levels, as well as the dilated ER lumen found by TEM, confirmed the occurrence of ERs after HFD feeding for more than six weeks.

There are a series of lipid-synthesis-related proteins bound at the ER membrane. Among them, SREBP-1C is a key transcription factor, which is responsible for activating the transcription of its downstream genes encoding rate-liming enzymes of fatty acid and triacylglycerol biosyntheses, such as FAS and ACC [31]. A recent study also indicates that the up-regulation of SREBP-1C impairs autophagic flux and thus suppresses fat degradation [32]. In the present study, we found that the expression of *srepb-1c* and *acc* were increased along with the activation of ERs. Liver fat accumulation is a complex process, which is determined by fat transportation, secretion, oxidation, and *do novo* synthesis [1]. Beyond lipid synthesis, some previous studies have demonstrated that ERs decreased hepatic fat oxidation and secretion [33]. Taken together, ERs play a role in excessive fat deposition, and targeting the inhibition of ERs may be an attractive therapeutic modality for fatty liver. 4-PBA is a short-chain fatty acid with an aromatic group, which has been proven to be a non-toxic pharmacological compound [34]. The United States Food and Drug Administration (FDA) has approved its clinical use [35]. There are many studies that have demonstrated that 4-PBA is a potent inhibitor of ERs, owing to the fact it can serve as a molecular chaperone to repair misfolding protein effectively [36,37,38,39]. Here, we added 50 mg/kg 4-PBA to an HFD to verify our conjectures. As expected, 4-PBA supplementation notably down-regulated the protein abundance of GRP78 and CHOP and eliminated the alterations of ultrastructure caused by an HFD, indicating ERs was suppressed. This is similar to the results obtained in previous mouse studies. 4-PBA can inhibit endoplasmic reticulum stress, reduce the content of endoplasmic reticulum stress markers, and alleviate the harm caused by a high-fat diet [15]. Additionally, 4-PBA administration reduced the liver, serum, and whole-body fat deposition, as well as the expression of *srebp-1c*, *fas,* and *acc*. 4-PBA can prevent the activation of UPR. Therefore, PERK, a key site of one of the three classical pathways of UPR, is also inhibited. Gene *srebp-1c* is regulated by upstream PERK [40], so its expression is down-regulated. This is strong support for our experimental results. It has been supported by the fact that the SREBP-1C/FAS/ACC pathway was a reliable regulation target of fat deposition in mammals and fish. Overall, our results confirmed that the inhibition of ERs down-regulated the SREBP-1C/FAS/ACC pathway, indicating ERs is an attractive target for the remission of excessive fat accumulation.

## 5. Conclusions

In conclusion, long-term (≥six weeks) HFD feeding caused a hepatic fat overload of *L. maculate*, which may be largely due to the activation of ERs. Then, ERs up-regulated the gene expression of the SREBP-1C/ACC/FAS pathway. 4-PBA as an ER stress inhibitor could reduce fat deposition caused by an HFD via regulating ER stress.

## Data Availability

Data are contained within the article and Supplementary Materials.

## Supplementary Materials

The following supporting information can be downloaded at: https://www.mdpi.com/article/10.3390/metabo12121197/s1, Table S1: Formulation and proximate composition of experimental diets; Table S2: Sequences of primers used for RT-qPCR.
