# Supplementary material for: 4-PBA Attenuates Fat Accumulation in Cultured Spotted Seabass Fed High-Fat-Diet via Regulating Endoplasmic Reticulum Stress
Source: Metabolites. 2022 Nov 30;12(12):1197. doi: 10.3390/metabo12121197 (PMC9784988; doi:10.3390/metabo12121197)
Supplement: Supplementary file 1 [file metabolites-12-01197-s001.zip › metabolites-1978859-supplementary.pdf]

Supplementary Material

Table S1. Formulation and proximate composition of experimental diets.

Ingredients (g/kg)	NFD	HFD
**Fish meal**	**268.5**	**268.5**
**Poultry meal**	**84.7**	**84.7**
**Wheat gluten**	**73**	**87.5**
**Soybean meal**	**250**	**250**
**α-starch**	**30**	**30**
**Wheat flour**	**201.1**	**125.1**
**Fish oil**	**19.3**	**50**
**Soybean oil**	**19.3**	**50**
**Soybean Lecithin**	**20**	**20**
**Premix1**	**13.5**	**13.5**
**Calcium biphosphate**	**20.8**	**20.8**
**Proximate composition (g/kg)**		
**Moisture**	**49.3**	**37.4**
**Protein**	**438.6**	**436.9**
**Lipid**	**110.7**	**168.6**
**Ash**	**85.7**	**84.8**

1Premix supplied the following minerals (g/kg) and vitamins (IU or mg/kg): $CuSO_4 \cdot 5H_2O$, 2.0 g; $FeSO_4 \cdot 7H_2O$, 25 g; $ZnSO_4 \cdot 7H_2O$, 22 g; $MnSO_4 \cdot 4H_2O$, 7 g; Na_2SeO_3, 0.04 g; KI, 0.026 g; $CoCl_2 \cdot 6H_2O$, 0.1 g; Vitamin A, 900000 IU; Vitamin D, 200000 IU; Vitamin E, 4500 mg; Vitamin K3, 220 mg; Vitamin B_1, 320 mg; Vitamin B_2, 1090 mg; Niacin, 2800 mg; Vitamin B_5, 2000 mg; Vitamin B_6, 500 mg; Vitamin B_{12}, 1.6 mg; Vitamin C, 5000 mg; Pantothenate, 1000 mg; Folic acid, 165 mg; Choline, 60000 mg.

Table S2. Sequences of primers used for RT-qPCR.

Target gene	Primer sequence (5'-3')	Amplification efficiency
β-actin1	**F: TCGAGCACGGTATTGTGACC**<br>**R: TCAGGTGCAACTCTCAGCTC**	**99.87%**
grp78	**F: GGGAGAAGAGGAGAAAGGTCTG**<br>**R: GCTTATCACCGCTCCGCTT**	**99.65%**
chop	**F: TGGGAGAAGAGGAGAAAGGTCT**<br>**R: CTTATCACCGCTCCGCTTGG**	**99.98%**
perk	**F: GTTTTCACCCCAGCAAGCAG**<br>**R: AACCTTAGTGTCGGCCTTGG**	**99.94%**
ire1	**F: AAAGGTTGTTCAGGGTGGCAT**<br>**R: GCAGCAATCAATCAACAAGCAAA**	**99.79%**
atf-6	**F: AACGAGCACTTGAGGAGAGC**<br>**R: CAGACGCTCACTCCTTGTGT**	**94.60%**
atf-4	**F: GTGTTGGTGCTTTGGGAACG**<br>**R: CATCAGACTCGCCCCTGTTT**	**99.79%**
eif-2α	**F: CTCTCCACACGAGGTGCTTC**<br>**R: TCGTGGTCTTCTGGGTGGTA**	**99.66%**
fas	**F: AAACTGAAGCCCTGTGTGCC**<br>**R: CACCCTGCCTATTACATTGCTC**	**98.80%**
srebp-1c	**F: CCTCACTCTGCAGCCAATCA**<br>**R: CGTAGTCCCACCCTCAAACC**	**99.48%**
acc	**F: AAGGCGGTGGTGATGGATTT**<br>**R: GGCCATGTCGCCTTTGTTTT**	**99.79%**

grp78: glucose regulated protein 78; *chop*: C/EBP homology protein; *perk*: PKR-like endoplasmic reticulum kinase; *ire1*: inositol-requiring enzyme 1; *atf-6*: activating transcription factor 6; *atf-4*: activating transcription factor 4; *eif-2α*: eukaryotic initiation factor 2; *fas*: fatty acid synthetase; *srebp-1c*: sterol-regulation element binding protein-1c; *acc*: acetyl-coa carboxylase.

1Reference gene
